# Quality of Life in Orthodontic Cancer Survivor Patients—A Prospective Case–Control Study

**DOI:** 10.3390/ijerph17165824

**Published:** 2020-08-12

**Authors:** Maria Mitus-Kenig, Marcin Derwich, Ewa Czochrowska, Elzbieta Pawlowska

**Affiliations:** 1 Department of Experimental Dentistry and Prophylaxis, Medical College, Jagiellonian University in Krakow, 31-008 Krakow, Poland; maria.mitus@interia.pl; 2 ORTODENT, Specialist Orthodontic Private Practice in Grudziadz, 86-300 Grudziadz, Poland; 3 Department of Orthodontics, Medical University of Warsaw, 02-091 Warsaw, Poland; info@czochrowska.com; 4 Department of Orthodontics, Medical University of Lodz, 90-419 Lodz, Poland; elzbieta.pawlowska@umed.lodz.pl

**Keywords:** quality of life, oral health-related quality of life, orthodontic treatment, cancer survivors

## Abstract

*Background*: The aim of the study was to compare the quality of life (QoL) of cancer survivors with a control group of healthy subjects before, during, and after the orthodontic treatment. *Methods*: Consecutive cancer survivors (40 people) who were looking for orthodontic treatment between 2008 and 2015 were enrolled into the study. Healthy orthodontic patients matched for age (±4 years), sex, and malocclusion served as controls. The 14-item version of the Oral Health Impact Profile was used to assess the effect of orthodontic treatment on QoL before, during, and after the orthodontic treatment. *Results*: There were no significant differences between both groups regarding the cast model, cephalometric analysis, and photographic documentation analysis. There was a significant worsening of QoL after the onset of the orthodontic treatment with a significant improvement after the treatment. Male cancer survivor patients reported significantly lower QoL during the treatment time, which was not observed in the male control group. *Conclusions*: The outcome of orthodontic treatment in cancer survivors did not differ from the healthy orthodontic patients. The orthodontic treatment had an impact on the oral health quality of life both in the cancer and the control groups with a significantly higher impact in male cancer survivor patients.

## 1. Introduction

The major cause of death among children worldwide is cancer [1,2]. The total number of childhood cancers constitutes approximately 2–3% of all cancers. Cancer occurs in more than 300,000 children every year worldwide. Prompt diagnosis, referral, and multidisciplinary care play significant role in successful cancer treatment [3]. Three most common childhood malignancies are: leukemias, brain tumors, and lymphomas [1,4]. Most of the childhood cancers may be cured with high survival rates, especially in high-income countries [3]. Low-income and middle-income countries are unfortunately characterized by diminished survival rates, mostly because of: misdiagnosis, inadequate access to treatment, death from toxicity, treatment abandonment, and relapse [5]. Despite the fact that the pediatric oncology is expensive, childhood cancer treatment is undoubtedly cost effective [6]. World Health Organization launched, in 2018, Global Initiative for Childhood Cancer, which aims to ensure the survival of at least 60% of children with cancer by 2030 [3].

Contemporary advances in cancer treatment resulted in 80% overall survival of young patients suffering from cancer. It is estimated that, at present, 1/900 young adults had undergone a successful cancer treatment in their childhood [7]. Therefore, the number of cancer survivors looking for orthodontic consultation and treatment might be growing. The orthodontic treatment in postoncological patients is expected to be much more complicated, because up to 50% of those patients have experienced short- and long-term adverse effects of both systemic and local oncologic treatment [8].

Radiotherapy may lead to oral mucositis, dysfunctional taste, malnutrition, and radiation caries [9]. Moreover, growing patients treated with chemotherapy and radiotherapy present the below mentioned side effects of oncological treatment: viscerocranium growth disorders, reduction in size of the alveolar processes of the maxilla and mandible, temporomandibular joint disorders (TMD), reduction of the length of pubertal growth, inhibition of tooth development, microdontia, hypodontia/oligodontia/anodontia, demineralization of the hard tissues of the teeth, shortening of dental roots—V-shaped roots, premature closing of root canal apices, root resorption, and tooth mobility [9,10].

According to the American Academy of Pediatric Dentistry (AAPD), there are three major objectives of a dental/oral examination after the end of oncological treatment (exclusive of HCT, hematopoietic cell transplantation). These are: to maintain optimal oral health, to reinforce to the patient/parents the importance of optimal oral and dental care for life, and to address and/or treat any dental issues that may arise as a result of the long-term effects of cancer therapy [11,12]. AAPD also presented a few suggestions regarding the orthodontic treatment after the oncological treatment has been finished. Orthodontic treatment may be commenced after at least 2 years of survival without any oncological disease and only in patients who are no longer taking immunosuppressive drugs. It is suggested to use appliances that minimize the risk of root resorption, to use lighter forces, to terminate treatment earlier than normal, to choose the simplest method for the treatment needs, and not to treat the lower jaw [11,12].

Although, the clinical examination of oral health is very important in the process of proper diagnosis and the accurate treatment planning, it does not include any functional or psychosocial aspects regarding the oral health. Therefore, the Oral Health-Related Quality of Life (OHRQoL) has been invented [13,14]. Among different indices meeting the criteria for OHRQoL, one of the most often used is the 14-item Oral Health Impact Profile (OHIP-14) [13].

The quality of life (QoL) of orthodontic patients has gained more attention in recent years. It is a multidimensional concept that includes subjectively perceived physical, psychological, and social functions, as well as a sense of subjective well-being in the areas of life that are important to individuals [15]. This assessment was introduced for better understanding of the impact of oral health on the daily lives of patients. However, the results of orthodontic treatment of cancer survivors and its influence on their quality of life remain unknown.

Therefore, the aim of the study was to compare the quality of life of cancer survivors with a control group of healthy subjects before, during, and after the orthodontic treatment.

## 2. Materials and Methods

### 2.1. Study Population

The study was held since January 2008 till December 2015. The study sample consisted of 40 consecutively selected patients (14 male and 26 female) with the history of childhood cancer who came to the specialist orthodontic practice, looking for orthodontic treatment. The median age of this group was 19.4 (range 14–28 years). Primarily, the study included 46 patients with the history of childhood cancer, but six patients were subsequently excluded from the study according to the exclusion criteria. The exclusion criteria were: previous orthodontic treatment, severe dentofacial deformities, such as cleft lip and palate, poor periodontal health, mental health disorders, including: anxiety disorders, depression, bipolar disorder, schizophrenia, and patients who did not agree to take part into the study.

The control group of 40 patients (14 male and 26 female) with a median age of 19.2 (range 14–28 years) was selected by matching age (±4 years), gender, and malocclusion. Healthy patients matched for age (±4 years), gender, and malocclusion served as control group.

We calculated the required sample size considering two equally numbered groups of patients, 90% of power, type I error 0.05, to detect 20% difference in OHIP-14 should include 175 patients. However, after analyzing 40 oncologic and control patients we did not see any difference between the groups.

All of the patients were recruited and treated in the same specialist orthodontic private practice in Krakow (Poland).

**Figure 1:**
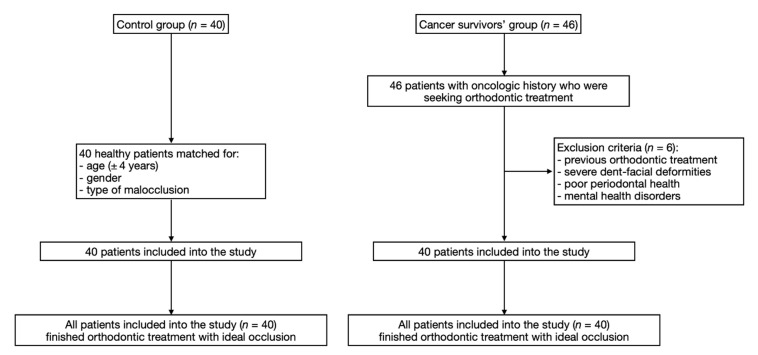
The flow chart of participation diagram.

Figure 1 presents the flow chart of participation. The baseline characteristics of cancer survivors’ and control group are presented in Table 1.

Table 2 presents the general characteristics of the cancer survivors’ group, including the type of the cancer, mean age at diagnosis, follow-up time, and treatment modality.

The study was approved by the Medical Board Ethical Committee (50/KBL/OIL/2010) and was conducted with the ethical principles of the World Medical Association Declaration of Helsinki. All of the patients received and signed informed consent. Parents received a letter describing the study protocol and requesting consent for their children to participate in the study.

### 2.2. Study Protocol

All the treatment and diagnostic records were prospectively collected in the database, according to the recognized clinical practice guidelines. All of the patients were treated orthodontically with fixed orthodontic appliances in both jaws.

The primary endpoint was to assess the quality of life of the patients. The secondary endpoints included: establishing a normal occlusion [16] (defined as class I molar and canine relations on both sides, normal occlusal contacts, midline symmetry, normal overjet and overbite, levelling and aligning the teeth within dental arches, improving/maintaining the aesthetics of the face and smile (assessed using the weighted Peer Assessment Rating–wPAR–Index), and avoiding complications during and after the treatment, defined as any event occurring in the follow-up period, which would require the treatment not routinely applied in this particular period).

In both groups, there were no aberrations in the root development diagnosed in the pantomographic X-ray before the onset of the orthodontic treatment.

The process of orthodontic diagnosis was performed by two independent certified specialists of orthodontics, whereas the orthodontic treatment was performed by the certified specialist of orthodontics with 18 years of experience in the field of orthodontics. All of the patients were treated orthodontically with the vestibular fixed appliances with the 0.022-in bracket slot and the MBT prescription. In particular, 90% of the examined patients were treated with no extractions, whereas the remaining 10% of the patients were treated with extractions, mostly because of severe crowding in lower dental arch. All of the cases were treated with sliding mechanics. The anchorage was increased by the usage of Goshgarian transpalatal bar and intermaxillary elastics. None of the treated cases required the usage of skeletal anchorage devices, including microscrews or miniplates.

All participants filled in the 14-item version of the Oral Health Impact Profile (OHIP-14), which is well-known and valid oral health-related quality of life indicator [15]. It was used to define seven conceptual dimensions of impact including: functional limitation (e.g., troubles while pronouncing words and dysfunctional taste), physical pain (e.g., painful aching and problems with eating), psychological discomfort (e.g., self-consciousness and tension), physical disability (e.g., unsatisfactory diet and interrupted meals), psychological disability (e.g., difficulty to relax and feeling of being embarrassed), social disability (e.g., irritability with others and irritability doing work), and handicap (e.g., unsatisfactory life and problems to function normally). Patients’ responses were rated on the 5-point Likert scale and were coded as: 0 (= never), 1 (= hardly ever), 2 (= occasionally), 3 (= fairly often), and 4 (= very often/every day) [13]. Table 3 presents the list of questions in the OHIP-14 Questionnaire.

Two different methods of scoring were used: the OHIP-14 additive method, in which the total score was calculated summing up the values for the 14 items (the total score ranges from 0 to 56), and OHIP-14 simple count, in which the total score was calculated summing up the number of domains reported as occasionally or more frequently (the number of domains ranges from 0 to 7). The OHIP-14 was evaluated before treatment, 2 weeks and 3 months after the onset of the treatment, and after the end of the treatment.

### 2.3. Statistical Analysis

The data were analyzed using Statistica 12.0 software (StatSoft Inc., Tulsa, OK, USA). No data was missing. Categorical variables were described as percentages of the total population, while continuous variables were reported as median and range. The Pearson’s chi-square or Fisher’s exact tests were applied, The Shapiro–Wilk and the Kolmogorov–Smirnov tests, with the Lilliefors correction, were used to confirm the normality of the distribution of the continuous variables. The unpaired Student *t* test or the nonparametric Mann–Whitney U tests were used for comparisons. The ANOVA test with repeated responses was used to assess differences in OHIP-14 across groups and time of treatment. Statistical significance was established at the level of *p* ≤ 0.05.

## 3. Results

This clinical trial was characterized by 81% statistical power, assuming two groups of 40 people each and RMSSE (root-mean square standardized effect) value 0.46.

The majority of the cancer survivors were diagnosed with leukemia (54%), followed by non-Hodgkin’s lymphoma (15.4%), Wilms tumor (15.4%), neuroblastoma (7.7%), and soft tissue sarcoma (7.7%). The median time from oncologic treatment was 9 years with a range from 6 to 12 years.

Due to matching process, 26 patients (65%) had skeletal class II, 9 patients (22.5%) had skeletal class I, and 5 patients (12.5%) had skeletal class III, both in the cancer survivors’ and the control group.

The average time of follow-up was 18 months. The average time of treatment in the cancer survivors’ group was significantly shorter than in the control group (12.5 vs. 18.0 months; *p* < 0.01). There were no significant differences in the outcomes of the orthodontic treatment achieved in both groups, assessed using study models, cephalometric analysis, and photographic documentation. Appropriate ideal occlusion was achieved in all patients with mean weighted PAR scores of 4.2–6.0 in both study groups. The reduction in the weighed PAR score was on average 81.7% and 80.5% in the control and cancer survivors’ group, respectively.

Ten cancer survivor patients and 2 patients from the control group had their orthodontic appliances temporarily removed to perform magnetic resonance imaging (MRI). Eight patients with ceramic brackets had MRI examination without removing the appliances. Significantly more patients from the cancer survivors’ group had oral mucositis (11 vs. 4; *p* < 0.05). Moreover, in the control group, there were two patients allergic to nickel. Three patients from the cancer survivors’ group had some signs of orthodontic root resorption (3 vs. 0; *p* < 0.05).

### 3.1. OHIP-14 Total Score

The orthodontic treatment had a statistically significant impact on the oral health-related quality of life in both groups. The OHIP-14 mean total score was significantly higher in both groups after 2 weeks (*p* < 0.001) and 3 months (*p* < 0.001) after the onset of the orthodontic treatment and significantly lower after the orthodontic treatment (*p* < 0.001) in comparison to the pretreatment results. However, when comparing both groups 2 weeks and 3 months after the onset of the orthodontic treatment, the cancer survivors’ group had higher values of the mean OHIP-14 total score, but the differences were not statistically significant.

**Figure 2:**
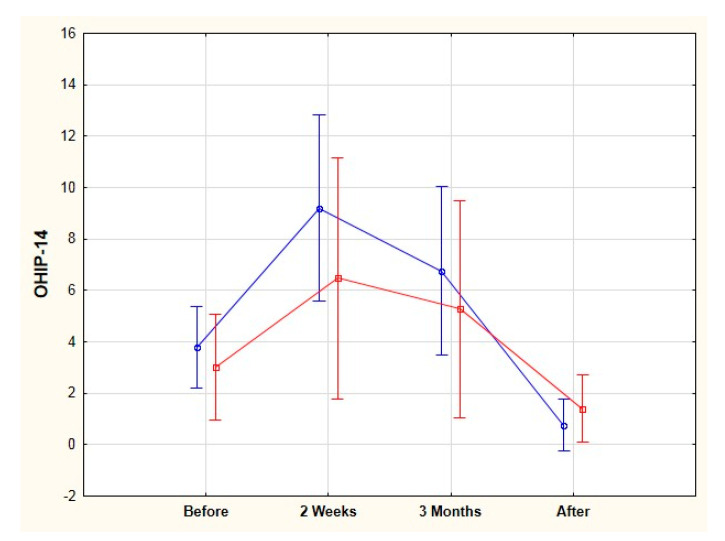
The 14-item Oral Health Impact Profile (OHIP-14) mean total score values before, during, and after orthodontic treatment in cancer survivors’ (blue line) and control (red line) groups.

Figure 2 presents the mean total score values of the OHIP-14 index before, during, and after the orthodontic treatment in cancer survivors’ and control groups.

Table 4 presents the OHIP-14 mean total score in the cancer survivors’ and control groups before, during, and after the orthodontic treatment.

Table 5 shows the mean scores of the OHIP-14 index for individual domains observed before the onset of the orthodontic treatment, 2 weeks after the onset of orthodontic treatment, 3 months after the onset of orthodontic treatment, and after finishing the orthodontic treatment. The only statistically significant differences between the examined groups referred to the psychological discomfort. The psychological discomfort 2 weeks and 3 months after the onset of the treatment was significantly higher in the cancer survivors’ group compared to the control group (*p* < 0.001).

Table 6 presents the probability values (p-values) describing the differences regarding the mean OHIP-14 scores between different stages of orthodontic treatment (before treatment vs. 2 weeks after the onset of the treatment, before treatment vs. 3 months after the onset of the treatment, and before treatment and after the treatment) in each domain, independently for the cancer survivors’ and control groups. The presented results indicate that orthodontic treatment had the highest impact on functional limitation and physical pain in both groups. Statistical significance of the differences regarding the mean OHIP-14 score in the domains psychological discomfort, physical disability, and psychological disability was higher for the cancer survivors’ group. Moreover, orthodontic treatment had no impact on social disability.

**Figure 3:**
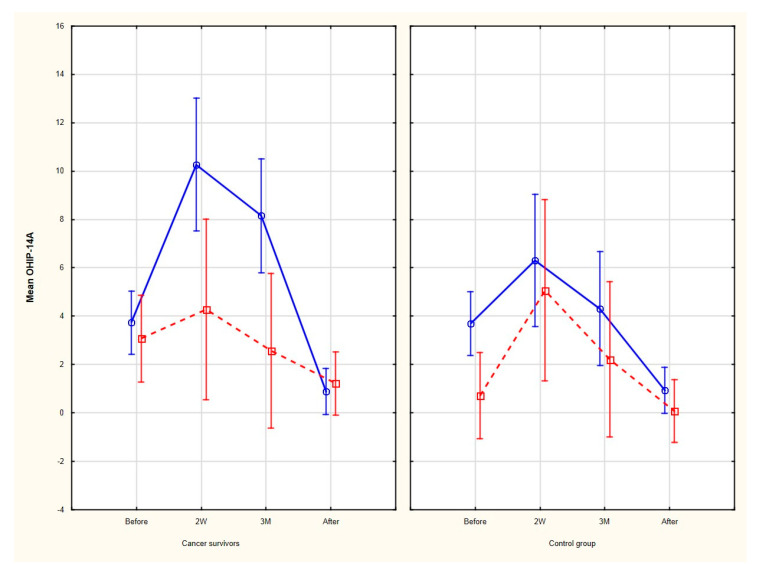
Gender differences in OHIP-14 total score in the cancer survivors’ and control groups (male patients are depicted with blue line and female patients with red dotted line). Here, 2W—2 weeks after the onset of the treatment, 3M—3 months after the onset of the treatment, and OHIP-14A—OHIP-14 index scored with the additive method.

When it comes to gender differences, male comparing to female cancer survivor patients reported a significantly lower quality of life during the treatment period (2 weeks and 3 months after the onset of the orthodontic treatment), which was not seen in the male control group (2 weeks: 10.3 ± 10.2 vs. 6.3 ± 5.7; 3 months: 8.2 ± 8.9 vs. 4.3 ± 4.5; *p* < 0.05, respectively). These differences were not observed before and after the treatment. Figure 3 presents the gender differences in the OHIP-14 total score between the cancer survivors’ and the control groups.

### 3.2. OHIP-14 Simple Count

Analyzing the number of patients with impaired domains, there were two statistically significant differences between the examined groups regarding only the psychological discomfort domain. The number of cancer survivor patients who reported psychological discomfort 2 weeks (13 vs. 2, *p* = 0.002) and 3 months (11 vs. 2, *p* = 0.006) after the onset of the orthodontic treatment was significantly higher than the number of patients form control group. Table 7 presents the detailed number of impaired domains in both groups the cancer survivors’ and the control ones.

There were no statistically significant differences between both groups regarding the number of subjects with oral health impacts calculated by adding the number of impacts reported as occasionally or more frequently. Table 8 presents the number of patients with oral health impacts measured on the basis of the OHIP-14 simple count.

## 4. Discussion

Contemporary oncologic treatment is predominantly multimodal and involves surgery, chemotherapy, and radiotherapy. There are numerous studies showing the influence of these methods of treatment on both the alveolar bone and the surrounding soft tissues, which results in growth restriction, development of an asymmetry, impaired occlusal development, and dental and root malformations [18,19,20]. The risk for these complications increases not only with the younger age of the patient (particularly in children undergoing oncologic treatment below the age of 5 years) but also with the exposure to high doses of chemotherapeutic agents and high dose radiotherapy [21].

This is the first study that prospectively assesses both the outcomes of the orthodontic treatment and the patients’ quality of life before, during, and after the orthodontic treatment between the cancer survivor and the control groups, which were perfectly matched for age, gender, and type of malocclusion.

According to our research, there were no statistically significant differences regarding the orthodontic treatment outcomes between cancer survivors’ group and healthy subjects. We observed a higher number of oral mucositis and root resorption in the cancer survivors’ group. However, early detection of those complications could have prevented their long-term adverse effects. Oral mucositis is a diffuse ulcerative lesion, associated with radiotherapy in the course of head and neck cancer treatment [22]. According to Sonis [22], there is nearly 100% risk of oral mucositis development in the standard chemoradiation protocols in the area of head and neck in patients who receive chemotherapy and radiotherapy with cumulative radiation doses higher than 30 Gy.

Several methods have been developed to measure people’s oral health-related quality of life. Hongxing et al. [13] compared the validity and reliability of two different indices: OHIP-14 and the Oral Impact of Daily Performance (OIDP). According to the authors, although both indices had reasonable validity and reliability, the OHIP-14 presented increased sensitivity towards less severe impacts. Montero-Martin et al. [23] validated the usage of the OHIP-14 among adult Spanish people. The authors concluded that the OHIP-14 was a precise, valid, and reliable index that can be used to assess quality of life related to oral health. Therefore, the OHIP-14 is one of the most commonly used questionnaires. This index was proven to be reliable, sensitive to changes and showing adequate cross-cultural consistency.

Andiappana et al. [15] noticed in their systematic review and meta-analysis regarding the association between the presence of malocclusion and patients’ quality of life that there was a high level of heterogeneity among the examined studies and the results varied between populations. Most of the patients were nononcologic ones, who had been qualified for orthodontic treatment or for orthognathic surgery. These studies revealed also that the patients with a malocclusion had a higher OHIP-14 total score in comparison to those without malocclusion [15]. Until now, there has been no study reporting the quality of life at the end of the active orthodontic treatment in postoncological treatment patients.

We have proven, that orthodontic treatment had an impact on the oral health quality of life in both groups: the cancer survivors’ and the control ones, by decreasing the quality of life during the treatment and increasing it after its completion. Moreover, the impact of orthodontic treatment on patients’ quality of life was significantly higher in male cancer survivor patients. Physical pain, psychological discomfort, and psychological disability domains contributed most significantly to higher values of the OHIP-14 index during the treatment. However, only psychological discomfort turned out to be significantly different between the cancer survivors’ and control groups. An interesting finding from our study is that cancer survivors’ male patients had a significantly worse quality of life during active treatment in comparison to the male controls. This effect was not seen in female cancer survivor patients. We can only hypothesize on the psychological influence of the previous disease history on the current treatment. The long-term positive and negative effects of post-traumatic stress disorder in childhood cancer survivors are well documented in the literature [24,25] and could be further studied on a larger group using more detailed questionnaires or psychological consultations.

Johal et al. [26] also noticed that the orthodontic treatment with fixed appliances had a negative impact on the OHIP-14 scores within the first 3 months after the onset of the orthodontic treatment. The OHIP-14 values after first and third month of active orthodontic treatment were significantly higher compared to the initial value of OHIP-14 index before treatment. Unlike, the authors found that there were no statistically significant differences regarding the OHIP-14 values before and after orthodontic treatment. However, most of the authors, who compared the OHIP-14 values before and after orthodontic treatment, found that the patients’ quality of life had significantly improved after orthodontic treatment [27,28,29,30]. Moreover, Zheng et al. [31] found that the positive impact of orthodontic treatment on the patients’ quality of life depends on the type of malocclusion. According to the authors, patients with class II benefitted the most from the space closure stage, whereas patients with class I malocclusion benefitted the most from alignment and levelling of their teeth, which is an initial stage of orthodontic treatment. Ni et al. [29] assessed the OHIP-14 in patients with skeletal class III, who had been treated surgically. The authors found that during the decompensation phase of orthodontic treatment, the patients’ quality of life had significantly decreased, whereas after the surgical orthodontic treatment, it had been significantly improved. The same observations presented Rezaei et al. [30].

There are researches which compared the OHIP-14 values between patients who had been and had not been treated orthodontically. According to the authors [32,33], patients who had been treated orthodontically had more positive oral health-related quality of life compared to the untreated subjects with malocclusion. The authors stated that dental malocclusion had significant negative impact on oral health-related quality of life. Kang et al. [34] also supported this thesis. Patients with either malocclusion or under the orthodontic treatment presented higher OHIP-14 scores comparing to the patients with normal occlusion or patients after the orthodontic treatment, who are in the retention phase.

There are some limitations to our study. The study population is relatively small and consists of cancer survivors treated for various malignancies, both solid tumors and hematologic malignancies. Moreover, the regimen used for oncologic treatment varies between patients making any comparison of its influence difficult. Finally, different types of orthodontic treatment biomechanics could have had an impact on patients’ quality of life, including different types of anchorage, usage of intermaxillary elastics, or placing additional hooks on the archwire.

## 5. Conclusions

The outcomes of the orthodontic treatment achieved in cancer survivors’ group did not differ significantly from the outcomes of the orthodontic treatment achieved in the group of healthy subjects. The orthodontic treatment had an impact on the oral health-related quality of life in both groups: the cancer survivors and the control group with a significantly higher impact on the male cancer survivor patients. The assessment of the quality of life during orthodontic treatment allows better understanding of patients’ needs and, therefore, better addressing the orthodontic care, particularly in specific domains, especially in cancer survivor patients.

## Figures and Tables

**Table 1:** Baseline characteristics of cancer survivors’ and control group of patients.

Factor	Cancer Survivors’ Group	Control Group	*p-*Value ^a^
Number of patients (n) (female/male ratio)	40 (26/14)	40 (26/14)	1.000
Median age (range) (years)	19.4 (14–28)	19.2 (14–28)	0.889
Orthodontic assessment (the same for both groups)	Skeletal class II: 26 patients each group Skeletal class I: 9 patients each group Skeletal class III: 5 patients each group		1.000

^a^ Mann–Whitney U test.

**Table 2:** General characteristics of the cancer survivors’ group, including the type of cancer, mean age at diagnosis, follow-up time, and treatment modality.

Diagnosis	No of Cases	Mean Age at Diagnosis	Follow-up Time	Treatment Modality	
				Chemotherapy	Radiotherapy
Leukemia	22	3.8 ± 1.3	9.5 ± 4.6	28	0
Neuroblastoma	3	0.7 ± 0.4	5.3 ± 1.1	4	0
Soft tissue sarcoma	3	2.2 ± 1.76	7.2 ± 2.0	4	1
Non-Hodgkin’s lymphoma	6	4.7 ± 2.1	8.0 ± 4.2	8	1
Wilms tumor	6	3.8 ± 2.2	6.1 ± 3.3	8	0

**Table 3:** The list of questions in the 14-item Oral Health Impact Profile (OHIP-14) Questionnaire [17].

The List of Questions in the OHIP-14 Questionnaire
**Functional Limitation**
Have you had trouble pronouncing any words because of problems with your teeth or mouth? Have you felt that your sense of taste has worsened because of problems with your teeth or mouth?
**Physical Pain**
3. Have you had painful aching in your mouth? 4. Have you found it uncomfortable to eat any foods because of problems with your teeth or mouth?
**Psychological Discomfort**
5. Have you been self-conscious because of your teeth or mouth? 6. Have you felt tense because of problems with your teeth or mouth?
**Physical Disability**
7. Has been your diet been unsatisfactory because of problems with your teeth of mouth? 8. Have you had to interrupt meals because of problems with your teeth or mouth?
**Psychological Disability**
9. Have you found it difficult to relax because of problems with your teeth or mouth? 10. Have you been a bit embarrassed because of problems with your teeth or mouth?
**Social Disability**
11. Have you been a bit irritable with other people because of problems with your teeth or mouth? 12. Have you had difficulty doing your usual jobs because of problems with your teeth or mouth?
**Handicap**
13. Have you felt that life in general was less satisfying because of problems with your teeth or mouth? 14. Have you been totally unable to function because of problems with your teeth or mouth?

**Table 4:** The OHIP-14 mean total score in the cancer survivors’ and control groups before, during, and after orthodontic treatment.

Time of Orthodontic Treatment (TX)	Cancer Survivors’ Group	Control Group	*p*-Value ^a^
	(mean ± SD) (Range)	(mean ± SD) (Range)	
Before TX	3.5 ± 3.9 ^1,2,3^	3.7 ± 3.0 ^4,5,6^	0.278
	(0–14)	(0–9)	
2 weeks after the onset of TX	8.2 ± 8.9 ^1^	8 ± 5.0 ^4^	0.159
	(0–32)	(0–20)	
3 months after the onset of TX	6.2 ± 7.9 ^2^	6.2 ± 3.9 ^5^	0.213
	(0–28)	(0–15)	
After TX	1.0 ± 2.5 ^3^	0.6 ± 2.3 ^6^	0.126
	(0–14)	(0–12)	

^1–6^ statistically significant differences with *p* < 0.001 (ANOVA); ^a^ Mann–Whitney U test; SD, standard deviation.

**Table 5:** The mean OHIP-14 score for individual domains observed before, during, and after orthodontic treatment for the cancer survivors’ and control groups.

OHIP-14 Domains	Functional Limitation	Physical Pain	Psychological Discomfort	Physical Disability	Psychological Disability	Social Disability	Handicap
Before (cancer survivors vs. control)	0.3 ± 0.5 0.3 ± 0.6	0.1 ± 0.3 0.1 ± 0.3	0.4 ± 0.7 0.2 ± 0.5	0.9 ± 0.9 0.6 ± 0.9	0.7 ± 1.3 0.5 ± 0.8	0.4 ± 0.7 0.3 ± 0.6	0.8 ± 1.1 0.6 ± 0.9
2 weeks (cancer survivors vs. control)	0.8 ± 0.9 0.9 ± 1.2	2.2 ± 2.7 1.6 ± 1.2	1.3 ± 2.0 ^1^ 0.3 ± 0.6 ^1^	1.7 ± 1.6 1.2 ± 1.0	1.2 ± 1.6 1.0 ± 1.3	0.4 ± 0.8 0.4 ± 0.7	0.7 ± 1.3 0.5 ± 0.8
3 months (cancer survivors vs. control)	0.4 ± 0.8 0.6 ± 0.9	1.6 ± 2.1 0.9 ± 0.9	1.0 ± 1.6 ^2^ 0.2 ± 0.9 ^2^	1.4 ± 1.7 0.8 ± 1.0	1.0 ± 1.5 0.7 ± 0.9	0.4 ± 0.8 0.2 ± 0.5	0.5 ± 1.2 0.4 ± 0.6
After (cancer survivors vs. control)	0.1 ± 0.3 0.1 ± 1.4	0.2 ± 0.5 0.1 ± 0.3	0.2 ± 0.6 0.1 ± 0.3	0.3 ± 0.6 0.1 ± 0.4	0.1 ± 0.5 0.1 ± 0.3	0.1 ± 0.4 0.1 ± 0.4	0.1 ± 0.3 0.1 ± 0.4

^1,2^ statistically significant differences with *p* < 0.001 (unpaired Student *t* test).

**Table 6:** The probability values (*p*-values) describing the differences regarding the mean OHIP-14 score between different stages of orthodontic treatment in each domain, independently for the cancer survivors’ and control groups.

OHIP-14 Domains	Functional Limitation	Physical Pain	Psychological Discomfort	Physical Disability	Psychological Disability	Social Disability	Handicap
Before vs. after	<0.001 *<0.001*	<0.001 *<0.001*	<0.001 *0.652*	<0.001 *<0.001*	<0.001 *0.004*	0.156 *0.342*	<0.001 *<0.001*
Before vs. 2 weeks	<0.001 *<0.001*	<0.001 *<0.001*	<0.001 *0.020*	<0.001 *0.225*	<0.001 *0.025*	0.656 *0.421*	0.034 *0.041*
Before vs. 3 months	<0.001 *<0.001*	<0.001 *<0.001*	<0.001 *0.030*	<0.001 *0.225*	0.041 *0.287*	1 *0.987*	0.019 *0.012*

All *p*-values were measured using ANOVA; the cancer survivors’ group and *the control group*.

**Table 7:** The number of patients with impaired domains in the cancer survivors’ and control groups.

OHIP-14 DOMAINS	Functional Limitation	Physical Pain	Psychological Discomfort	Physical Disability	Psychological Disability	Social Disability	Handicap
Before (cancer survivors vs. control)	1 3	0 0	4 1	12 10	8 6	6 2	8 7
2 weeks (cancer survivors vs. control)	6 11	16 17	13 ^1^ 2 ^1^	22 19	13 11	5 4	6 3
3 months (cancer survivors vs. control)	2 7	14 7	11 ^2^ 2 ^2^	16 12	12 6	5 3	5 3
After (cancer survivors vs. control)	1 1	2 1	3 1	2 1	2 1	2 2	1 2

^1^ statistically significant difference with *p* = 0.002 (Chi-square test), ^2^ statistically significant difference with *p* = 0.006 (Chi-square test).

**Table 8:** OHIP-14 simple count—the number of patients with oral health impacts.

Time of Orthodontic Treatment (TX)	Cancer Survivors’ Group	Control Group	*p-*Value
			(Chi-Square Test)
Before	6 (15.0%)	3 (7.5%)	0.289
2 weeks	13 (32.5%)	11 (27.5%)	0.716
3 months	11 (27.5%)	6 (15.0%)	0.172
After the treatment	2 (5.0%)	1 (2.5%)	0.556
